# Metabonomics Study of Ginseng Glycoproteins on Improving Sleep Quality in Mice

**DOI:** 10.1155/2019/2561828

**Published:** 2019-03-03

**Authors:** Ying Wang, Difu Zhu, Yinghong Chen, Ruizhi Jiang, Hong Xu, Zhidong Qiu, Da Liu, Haoming Luo

**Affiliations:** ^1^School of Pharmacy, Changchun University of Chinese Medicine, Changchun 130117, China; ^2^Jilin Academy of Chinese Medicine and Material Medica Science, Changchun 130012, China; ^3^Jilin Jice Inspection Technology Co., Ltd., Changchun 130117, China

## Abstract

The changes of brain metabolism in mice after injection of ginseng glycoproteins (GPr) were analyzed by gas chromatography mass spectrometry- (GC/MS-) based metabolomics platform. The relationship between sedative and hypnotic effects of ginseng glycoproteins and brain metabolism was discussed. Referring to pentobarbital sodium subthreshold test, we randomly divided 20 mice into two groups: control and ginseng glycoproteins group. The mice from the control group were treated with normal saline by i.p and GPr group were treated with 60 mg/kg of GPr by i.p. The results indicated that GPr could significantly improve the sleep quality of mice. Through multivariate statistical analysis, we found that there were 23 differential metabolites in whole brain tissues between the control group and the GPr group. The pathway analysis exhibited that GPr may be involved in the regulation of the pathway including purine metabolism, nicotinate and nicotinamide metabolism, glycine, serine and threonine metabolism, arginine and proline metabolism, alanine, aspartate and glutamate metabolism, and steroid hormone biosynthesis. This work is helpful to understand the biochemical mechanism of GPr on promoting sleep and lay a foundation for further development of drugs for insomnia.

## 1. Introduction

Dyssomnia is one of the most common health problems, involving difficulty getting to sleep, remaining asleep, or of excessive sleepiness. Generally, dyssomnias are characterized by a disturbance in the amount, quality, or sleep time. The reasons and mechanisms of dyssomnia are complicated, which may be a symptom of other primary medical diseases and mental illness or caused by the use, abuse, or exposure of certain substances. Many studies have suggested that dyssomnia is not only linked to mental disorders, including depression [1], but also to endocrine disorders (e.g., obesity, diabetes mellitus) and cardiovascular disorders (e.g., hypertension, heart disease) [2–6].

In recent years, a variety of advanced technology and new methods are used to study the biochemical mechanisms of disease. Metabolomics is a high-sensitivity, high-throughput profiling method to study the characteristic changes in low-molecular-weight metabolites [7]. Such an approach provides a comprehensive view of the changes in several metabolic and signaling pathways and their interactions [8]. Since genomics, epigenetics, transcriptomics, and proteomics all converge at the level of metabolomics and changes in metabolite concentrations are more substantial and defining than signals in other “omics”, metabolomics is considered to be the integration of all “omics”. Therefore, it is more reliable, sensitive, and powerful in reflecting changes in biological functions due to either disease or drug action [9, 10]. Metabolomics is also applied to sleep disorders recently [11].

Until now, metabolomics is widely used in traditional Chinese medicine. For example, Gao et al. used metabolomics to analyze the serum metabolites of galangin of many TCM herbs to elucidate its treatment effect on an experimental cerebral ischemia rat model [12]. Panax ginseng C.A. Mey has been used as traditional Chinese medicine (TCM) for more than one thousand years. Our previous reports showed that the ginseng glycoproteins had significant effects on analgesic and enhancing memory activity [13–15], moreover, which is also effective in improving sleep quality. Therefore, in this work, the pentobarbital sodium subthreshold test was used to study the effects of ginseng glycoproteins on sleep disorders. Subsequently, in order to understand the biochemical mechanisms of the sedative-hypnotic effects, metabolomics profiling was used to analyze the brain metabolic changes in mice following the intraperitoneal injection of ginseng glycoproteins, and the metabolic pathways related to sedative-hypnotic were discussed, which may be helpful for the future development of drugs based on ginseng glycoproteins for dyssomnia treatment.

## 2. Materials and Methods

### 2.1. Animals and Tissue Sample Collection

The ginseng glycoproteins sample (GPr) was prepared in our previous study [15].

This study was conducted in accordance with the National Institute of Health Guide for the Care and Use of Laboratory Animals and approved by the Institutional Animal Care and Use Committee of Jilin Academy of Chinese Medicine and Material Medica Science. Male mice (21–25 g) were used in the study. The animals were housed in a 12-h light 12-h dark cycle with food and water available ad libitum. All experimental animal procedures were performed according to the approval from the animal experimentation ethics.

Mice were randomly divided into two groups: control and GPr group. Each group consisted of 10 animals. The mice from the control group were treated with normal saline by intraperitoneal injection (i.p.), and the GPr group were treated with 60 mg/kg of GPr by i.p. After the last administration for 30 min, each animal was treated with 45mg/kg (hypnotic subthreshold dose, namely, the maximum dose of 90%~100% mice without sleep) of pentobarbital sodium by i.p. Sleep latency and sleep time were recorded in each group (the period of righting reflex disappeared more than 1 min was considered to fall asleep). The animals were subsequently sacrificed and the brains were rapidly removed, washed by normal saline, then placed in liquid nitrogen for 20 min, and kept at −80°C until the time of the assay.

### 2.2. Tissue Preparation

The whole brain tissues were taken into the 2 mL EP tubes and extracted with 0.45 mL extraction liquid (*Vmethanol*:* VChlorofrom*= 3:1), with vortex mixing for 30 s. They were homogenized in ball mill for 4 min at 45 Hz and then ultrasound treated for 5min (incubated in ice water), repeated for two times, and centrifuged for 15 min at 13 000 rpm. The supernatant was dried in a vacuum concentrator without heating. Then we added 80 *μ*L of Methoxyamination hydrochloride (20 mg/mL in pyridine), incubated for 30 min at 80°C, and added 100 *μ*L of the BSTFA regent (1% TMCS, v/v) to the sample aliquots, incubated for 1.5 h at 70°C.

### 2.3. GC-MS

Gas Chromatography Tandem Time-of-Flight Mass Spectrometry (GC-TOF-MS) is the basis of metabonomic data analysis [16, 17]. GC-TOF-MS analysis was performed using an Agilent 7890 gas chromatograph system coupled with a Pegasus HT time-of-flight mass spectrometer. The system utilized a DB-5MS capillary column coated with 5% diphenyl cross-linked with 95% dimethylpolysiloxane (30 m×250 *μ*m inner diameter, 0.25 *μ*m film thickness; J&W Scientific, Folsom, CA, USA). 1 *μ*L aliquot of the analyte was injected in splitless mode. Helium was used as the carrier gas, the front inlet purge flow was 3 mL/min, and the gas flow rate through the column was 1 mL/min. The initial temperature was kept at 50°C for 1min, then raised to 310°C at a rate of 10°C/min, and then kept for 8min at 310°C. The injection, transfer line, and ion source temperatures were 280, 270, and 220°C, respectively. The energy was -70 eV in electron impact mode. The mass spectrometry data were acquired in full-scan mode with the m/z range of 50-500 at a rate of 20 spectra per second after a solvent delay of 6.04 min.

### 2.4. Data Analysis

Chroma TOF 4.3X software of LECO Corporation and LECO-Fiehn Rtx5 database were used for raw peaks exacting, the data baselines filtering and calibration of the baseline, peak alignment, deconvolution analysis, peak identification, and integration of the peak area [18]. The statistical analysis was performed using software package (V14.1, MKS Data Analytics Solutions, Umea, Sweden). Principal component analysis (PCA) [19] and OPLS-DA were performed to visualize the metabolic differences between control and GPr groups. PCA showed the distribution of origin data. In order to obtain a higher level of group separation and get a better understanding of variables responsible for classification, supervised orthogonal projections to latent structures-discriminate analysis (OPLS-DA) were applied [20]. R^2^Y is the explained variation in Y; Q^2^ is the predicted variation. The range of both parameters is between 0 and 1; the closer they approach 1, the better they can be predicted or explained [21]. Variable importance was measured by the variable influence on projection (VIP). The VIP values exceeding 1.0 were first selected as changed metabolites. In step 2, the remaining variables were then assessed by Student's* t*-test (*P*≤0.05), and variables were discarded between two comparison groups. Metabolic pathway analysis was performed by inputting discriminant molecules into Metaboanalyst (available online at http://www.metaboanalyst.ca) and network analysis was processed by Metscape 3.1-based on Cytoscape 3.3.0.

## 3. Results

### 3.1. Effects of Subthreshold Doses of Pentobarbital Sodium on Sleep in Mice

SPSS10.0 software was used for statistical analysis. The difference between control group and GPr group was significant (P<0.05). The results showed that the intraperitoneal injection of ginseng glycoproteins at a dose of 60 mg/kg could significantly reduce the sleep latency (P<0.05) and prolong the sleep time of mice (P<0.01), as shown in Table 1.

### 3.2. GC-MS Profiling

**Figure 1:**
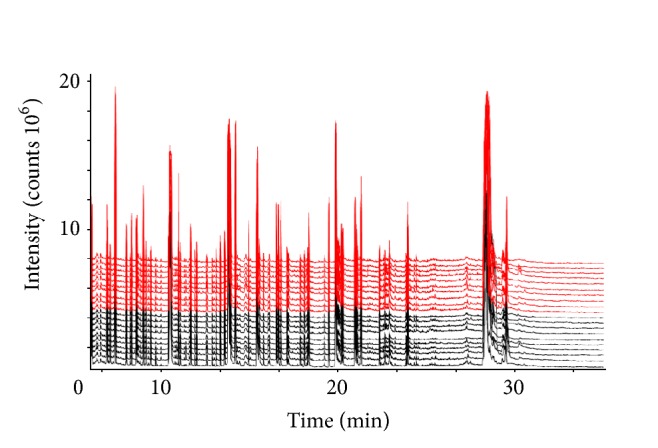
Typical GC/MS chromatograms of extracts from brain tissue of 20 mice. Top 10 lines (red): GPr group and the other 10 lines (black): control group.

GC-MS analysis of all the brain tissue extracts in the control and GPr groups revealed a large number of peaks as shown in Figure 1. In the chromatogram, the retention time and the corresponding intensity of the samples of control group and GPr group were reproducible. It is proved that the preprocessing and instrumental analysis systems adopted in this experiment both are stable and reliable. 284 peaks were authentically identified by comparing the mass spectrum of the peak with that available in the libraries and that of the reference compounds. These included amino acids, small organic acids, fatty acids, and lipids.

### 3.3. Statistical Analysis

**Figure 2:**
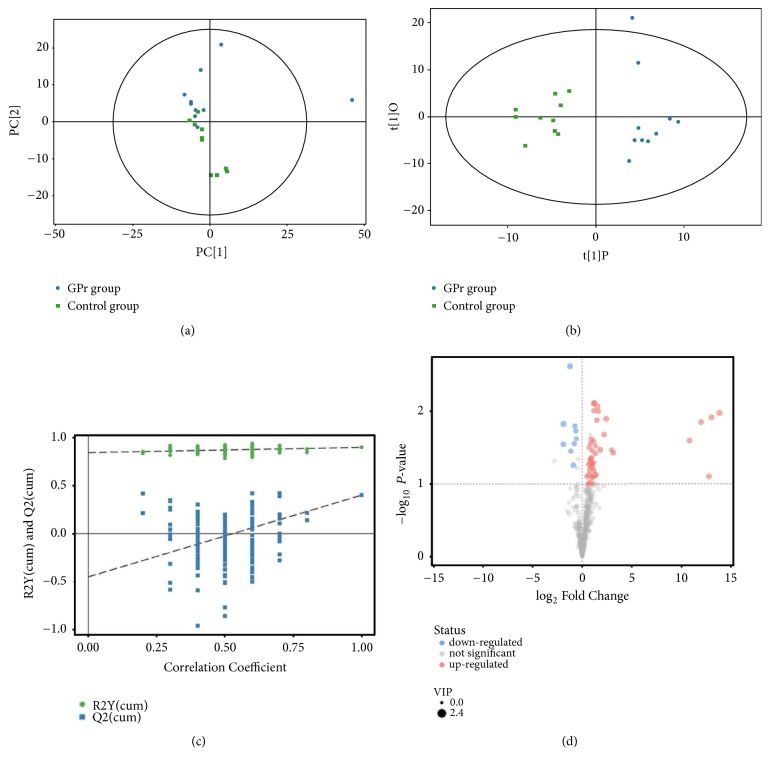
Score scatter plots of PCA and OPLS-DA model for two groups. (a) PCA, (b) OPLS-DA, (c) permutation test of OPLS-DA model, and (d) volcano plot.

Principal component analysis (PCA) could show the distribution of origin data in the statistical analysis. The score scatter plots of PCA (Figure 2(a)) showed that the control group and the GPr group of samples were basically in the confidence interval of 95%, which indicated that the results of two groups were credible. Additionally, in order to obtain a higher level of group separation and get a better understanding of variables responsible for classification, supervised orthogonal projections to latent structures-discriminate analysis (OPLS-DA) were applied. In the scatter plots of the OPLS-DA (Figure 2(b)), the control group and the GPr group could be separated distinctly, which indicates that there is a significant difference between the two groups in the metabolic level. It can also be seen that the points between the groups are scattered, which shows that the data not only have intergroup differences but also intra-group differences. Afterwards, the parameters for the classification from the software were R^2^Y=0.898 and Q^2^=0.401, which were stable and good to fitness and prediction. 7-fold cross validation was used to estimate the robustness and the predictive ability of our model; such permutation test was proceeded in order to further validate the model. The R^2^ and Q^2^ intercept values were 0.84 and -0.45 after 200 permutations. The low values of Q^2^ intercept indicate the robustness of the models and thus show a low risk of over fitting and reliable. Based on the OPLS-DA, a loading plot was constructed, which showed the contribution of variables to difference between two groups (Figure 2(c)). Volcano plots could be very intuitive and reasonable to screen out the differences between two groups, which was the visualization of the differential metabolites. In the volcano plots, each point represents a metabolite. The colors of scattered points represent the final screening results: the significantly upregulated metabolites were red, the downregulated metabolites were blue, and the nonsignificantly different metabolites were gray, as shown in Figure 2(d).

**Figure 3:**
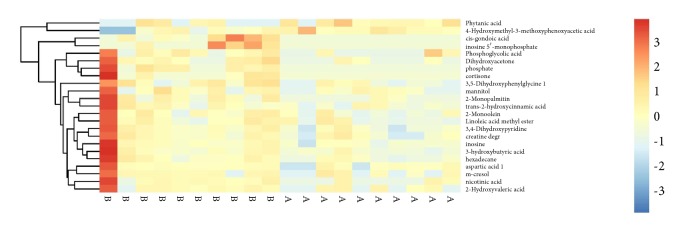
Heatmap visualization of the differential metabolites in response to treatment with GPr by i.p. Color denotes the abundance of metabolites, from the highest (red) to the lowest (blue).

On the basis of the OPLS-DA results, a total of 23 differential metabolites were identified, including amino acids, fatty acids, nucleosides, and organic acids (Table 2). After treatment of GPr, the levels of 4-Hydroxymethyl-3-methoxyphenoxyacetic acid and phytanic acid were reduced, and other 21 metabolites were increased. To intuitively inspect the tendencies in the variation of metabolite concentrations between the control and GPr groups, we produced a heatmap of differential metabolites according to the relative quantities of each marker. In comparison with the control, as shown in Figure 3, a total of 21 metabolites were up regulated (red) and a total of 2 metabolites were downregulated (blue) in the cold group, as listed in Table 2.

### 3.4. Pathway Analysis

**Figure 4:**
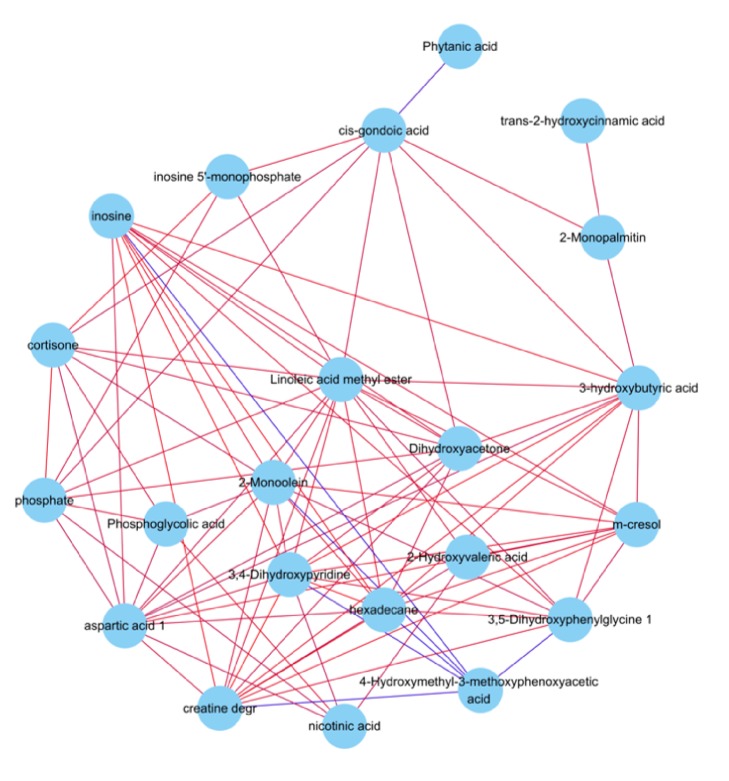
Correlation network analysis of differential metabolites. Each circle represents a metabolite, whose links indicate a significant correlation between the metabolites. Red lines indicate positive correlation and blue lines indicate negative correlation.

The correlation between 23 differential metabolites was calculated by Pearson correlation coefficient, as shown in Figure 4. Furthermore, all the pathways of the differential metabolite mapped were sorted out through the database. The pathway impact analysis suggested that GPr produced a significant impact on 5 metabolic pathways, including carbon metabolism (involved 3 differential metabolites), purine metabolism (involved 2 differential metabolites), glycine, serine and threonine metabolism (involved 2 differential metabolites), nicotinate and nicotinamide metabolism (involved 2 differential metabolites), and ABC transporters (involved 2 differential metabolites), as shown in Table 3.

**Figure 5:**
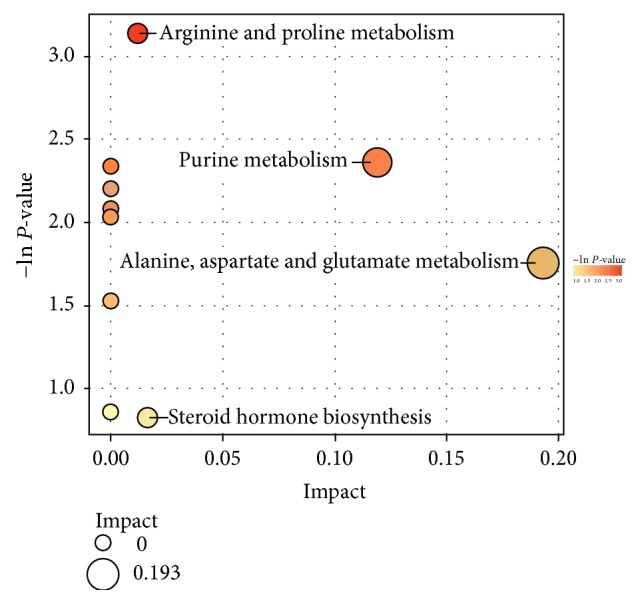
Pathway impact analysis for control group and GPr group, including alanine, aspartate and glutamate metabolism, purine metabolism, arginine and proline metabolism, and steroid hormone biosynthesis.

The metabolic pathways with significant difference between two groups were further identified by enrichment analysis and topological analysis. The results of metabolic pathway analysis are presented in the form of bubble diagrams in which each bubble represents a metabolic pathway (Figure 5). The abscissa and bubbles size indicated the impact factors in topological analysis, and the larger the size, the greater the impact factors; the ordinates and bubbles color indicated the P value of enrichment analysis, and the deeper the color, the smaller the P value and the more significant the degree of enrichment. The critical value of impact factors of metabolic pathway value was set to 0. The metabolic pathways with the value of impact factors greater than 0 were recognized as potential target pathways, including alanine, aspartate and glutamate metabolism, purine metabolism, arginine and proline metabolism, and steroid hormone biosynthesis. In particular, among them, the metabolic pathway of alanine, aspartate, and glutamate had the highest impact factor in topological analysis, while metabolic pathway of arginine and proline had the highest p value for enrichment analysis, which indicated that the difference of these two metabolic pathways between control and GPr group was the most significant.

## 4. Discussion

Sleep is controlled and regulated by brain [22]. In the present study, 23 differentially changed molecules in brain were identified. So the ginseng glycoproteins produced effect by changing the contents of these molecules. And all of these molecules could be the biomarkers for further tests of the glycoproteins. Through database analysis, five most closely pathways related to the metabolites were identified, some of which may affect sleep, mainly including purine metabolism, glycine, serine and threonine metabolism, nicotinate, and nicotinamide metabolism. In addition, the bubble diagram also points out that ginseng glycoproteins imposed a significant impact on 4 metabolic pathways including arginine and proline metabolism, purine metabolism, alanine, aspartate and glutamate metabolism, and steroid hormone biosynthesis.

### 4.1. Purine Metabolism

The results indicate that the change in purine metabolism pathway may have an effect on sleep quality by intraperitoneal injection of GPr. Adenosine and adenosine triphosphate (ATP) are the only purines known to play a role in cell-to-cell communication. They mediate many biological effects through cell-surface receptors (purine receptors) in extracellular matrix. Adenosine is a metabolite of ATP, which mainly accumulates in the extracellular domain and is widely distributed in the brain. In 1954, Fredberg and Sherwood first discovered that adenosine could promote sleep in cats, and then a large number of studies supported this viewpoint [23]. Adenosine, derived from the breakdown of ATP, accumulates during awakening and decreases as sleep progresses and so is considered as an endogenous sleep regulator. The adenosine receptor (AR1) activated could inhibit excitatory conduction in neurons, which is considered to be the most important receptor. Inosine and Inosine 5′-monophosphate (IMP) were found to be upregulated, which were also ones of the products of purine metabolism.

### 4.2. Nicotinate and Nicotinamide Metabolism

Nicotinic acid, also known as vitamin B3, one of the thirteen essential vitamins for the human body, could be transformed into nicotinamide in vivo. As the major precursor of the coenzyme nicotinamide adenine dinucleotide (NADH/NAD+), nicotinamide is crucial to life. In cells, it participates in the synthesis of NAD+ and NADP+, which are coenzymes involved in a wide variety of enzymatic oxidation-reduction reactions for energy production and, glycolysis, tricarboxylic acid cycle (TCA cycle), and the electron transport chain are the most notable events of these. Studies suggest that the expression of deacetylase Sirtuin type 3 (SIRT3) NAD+-dependent was significantly decreased in response to long-term sleep deprivation [24]. In GPr group, the content of nicotinate was upregulated, and it suggests that there is a close relationship between nicotinate and nicotinamide metabolism and sleep, which needs further study.

### 4.3. Glycine, Serine and Threonine Metabolism, Arginine and Proline Metabolism, Alanine, Aspartate, and Glutamate Metabolism

The glycine, serine, and threonine metabolic pathway has been thought to provide a major energy metabolism precursor substance for TCA cycle. Glycine is a simple, nonessential amino acid, which is involved in the production of phospholipids and collagen and releases energy as well. Glycine is also known as an inhibitory neurotransmitter, which possessed the ability to combine correlative NMDA receptor antagonist so as to exert an antidepressant effect. Arginine and proline metabolism is one of the central pathways for the biosynthesis of the amino acids arginine and proline from glutamate. Arginine is a precursor of nitric oxide (NO) biosynthesis, which can be converted into NO by nitric oxide synthase (NOS)[25]. More and more evidence suggests that NO, as a ubiquitous gaseous cellular messenger, plays significant roles in a variety of neurobiological processes. This regulatory molecule has many functions in the nervous system and in the process of endothelium-dependent vasodilatation [26]. The result was in accordance with our previous report, which indicated the sedative-hypnotic effect of ginseng may was involved in NO synthesis [13].

### 4.4. Steroid Hormone Biosynthesis

Steroid hormone acts as a hormone, which can be grouped into two classes: corticosteroids (typically made in the adrenal cortex) and sex steroids (typically made in the gonads or placenta). Steroid hormones help control metabolism, inflammation, immune functions, salt and water balance, development of sexual characteristics, and the ability to withstand illness and injury. Furthermore, later studies have shown that these steroids can also be synthesized and metabolized by cholesterol in the central and peripheral nervous systems independently. These steroids playing a role in the nervous system were called “neurosteroids”. The neurosteroids exist in the form of conjugates, whose concentration in the brain is much higher than that in the plasma, including pregnenolone (PREG), pregnenolone sulfate (PS), allopregnanolone (AP), dehydroepiandrosterone (DHEA), dehydroepiandrosterone sulfate(DS), and pregnenolone (PROG). Conventional wisdom holds that neurosteroids activate transcription factors and regulate gene expression by binding to intracellular receptors. It was found that the steroids also play a role through interaction with various neurotransmitter receptors on the cell membrane. Many studies suggest that neurosteroids act on GABAA receptor. For example, PROG and AP have been proved to be agonists of GABAA receptors, which are similar to barbiturate and can increase the influx of chloride and enhance the inhibition of GABA [27]. This may be one of the reasons why some neurosteroids can rapidly change the excitability of nerve cells and provide a possible mechanism for the hypnotic effect of neurosteroids.

## 5. Conclusions

In summary, we reported here that after i.p. injection of ginseng glycoproteins in mice caused a significant shift in metabolite profiles in brain. We also successfully used metabolomics approach to identify a number of metabolic pathways using the whole brain tissues of mice. The results suggested that the sedative-hypnotic actions of ginseng glycoproteins are associated with brain. This work not only opens up a new way to study the biochemical mechanism of traditional Chinese medicine but also provides the research basis for the development of ginseng glycoproteins-based drugs for the treatment of sleep disorders.

## Figures and Tables

**Table 1:** Effect of ginseng glycoproteins on the sleep of mice induced by pentobarbital sodium ($\overset{-}{x}\pm s,n=10$) (s).

Group	Sleep latency	Sleep time
Control	533.2 ± 110.3	3166.1 ± 1325.2
Ginseng glycoproteins	308.6 ± 68.3^*∗*^	5445.5 ± 1415.2^*∗∗*^

Compared with control group, ^*∗*^*P* < 0.05; ^*∗∗*^*P* < 0.01.

**Table 2:** The relative concentrations of differential metabolites in response to treatment with GPr by intraperitoneal injection in mice. Similarity value (SV) was used to evaluate the accuracy of the discriminating metabolite. Variable importance in the projection (VIP) was obtained from OPLS-DA with a threshold of 1.0. p-Value was calculated from Student's t-test. The red and blue arrows indicate up and down trends.

Metabolites	SV	Control	GPr	VIP	P-VALUE	Trend
Inosine	933	230	0.26883496	0.474806953	1.403129	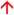
Aspartic acid 1	930	232	1.394412343	2.302817461	1.95754	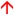
3-Hydroxybutyric acid	896	147	0.03413712	0.057421069	1.790805	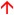
Phosphate	826	373	9.58593E-07	0.003834269	2.238158	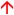
2-Monopalmitin	761	218	0.000916024	0.007080175	1.305231	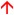
2-Monoolein	725	103	0.007766589	0.0193021	1.57449	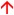
cis-Gondoic acid	697	175	9.58593E-07	0.013856645	2.387345	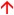
Inosine 5′-monophosphate	687	315	9.58593E-07	0.001733039	2.083737	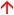
Nicotinic acid	586	180	0.001234995	0.004313206	2.41329	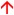
Dihydroxyacetone	558	163	0.000153415	0.000706533	1.70357	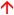
Linoleic acid methyl ester	542	86	0.022472983	0.055198241	1.340144	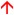
m-Cresol	534	165	0.001404886	0.002949009	1.930035	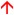
Mannitol	517	319	0.001031488	0.002153881	1.063901	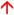
Cortisone	499	103	9.58593E-07	0.006679172	1.942847	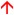
2-Hydroxyvaleric acid	446	102	0.000877641	0.001813946	1.95695	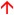
Phosphoglycolic acid	425	299	0.005146486	0.013489075	1.927157	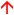
Phytanic acid	357	159	0.004245082	0.001927866	1.541776	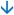
3,4-Dihydroxypyridine	337	256	0.003866274	0.007287569	1.81441	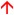
3,5-Dihydroxyphenylglycine 1	316	283	0.003695243	0.010222014	1.836686	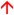
Hexadecane	279	199	0.017033444	0.030171359	2.100437	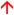
Creatine degr	276	316	0.000734181	0.001345046	1.841828	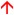
4-Hydroxymethyl-3-methoxyphenoxyacetic acid	241	366	0.001093006	0.000727609	1.032588	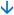
trans-2-Hydroxycinnamic acid	226	219	0.00088091	0.001805154	1.16949	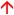

**Table 3:** The relative 5 pathways of differential metabolitein response toGPr.

pathway	Differential metabolites in the pathway
Carbon metabolism	Aspartic acid 1; Dihydroxyacetone; Phosphoglycolic acid
Purine metabolism	Inosine; Inosine 5′-monophosphate
Glycine, serine and threonine metabolism	Aspartic acid 1;Creatine
Nicotinate and nicotinamide metabolism	Aspartic acid 1;Nicotinic acid
ABC transporters	Aspartic acid 1;Phosphate

## Data Availability

The data used to support the findings of this study are available from the corresponding author upon request.

## Supplementary Materials

Supplementary Materials The file named “Models Information”mainly includes the number of multivariate statistical analysis models, model type (PCA, OPLS-DA), number of principal components of a model, the number of observations in the model, and other related information. The file named “Mean” mainly includes the information of metabolites, like the unique data number of the substance in this qualitative analysis, the matching degree between the substance and the material in the standard library, retention time, the number of substances detected in all experimental groups, and other related information. The file named “Differentially Expressed Metabolites” mainly includes the information on differential metabolites and screening conditions for differential metabolites in this experiment are VIP>1 and P-Value<0.1. The information contained in the document includes material name, the matching degree between the substance and the material in the standard library, retention time, the importance of the substance variable projection in the OPLS-DA model of this group, P-VALUE, Q-VALUE, and other related information. The file named “Metabolite Mapping” mainly includes the matching of differential metabolites in pathway analysis. It contained the name of matching substance, ID of the substance in the HDMB database, ID of the substance in the PubChem database, ID of the substance in the KEGG database, and matching situation. The file named “Pathway Analysis” mainly includes the information of related metabolic pathways. It contained metabolic pathway name, the number of metabolites in this pathway, number of different metabolites in the pathway, P value of metabolic pathway enrichment analysis, and other related information.
